# Convergence of Inflammatory Pathways in Allergic Asthma and Sickle Cell Disease

**DOI:** 10.3389/fimmu.2019.03058

**Published:** 2020-01-24

**Authors:** Amali E. Samarasinghe, Jason W. Rosch

**Affiliations:** ^1^Division of Pulmonology, Allergy-Immunology, and Sleep, Department of Pediatrics, College of Medicine, University of Tennessee Health Science Center, Memphis, TN, United States; ^2^Department of Microbiology Immunology and Biochemistry, College of Medicine, University of Tennessee Health Science Center, Memphis, TN, United States; ^3^Children's Foundation Research Institute, Memphis, TN, United States; ^4^Department of Infectious Diseases, St. Jude Children's Research Hospital, Memphis, TN, United States

**Keywords:** sickle cell disease (SCD), asthma, acute chest syndrome (ACS), respiratory infection, pulmonary inflammation

## Abstract

The underlying pathologies of sickle cell disease and asthma share many characteristics in terms of respiratory inflammation. The principal mechanisms of pulmonary inflammation are largely distinct, but activation of common pathways downstream of the initial inflammatory triggers may lead to exacerbation of both disease states. The altered inflammatory landscape of these respiratory pathologies can differentially impact respiratory pathogen susceptibility in patients with sickle cell disease and asthma. How these two distinct diseases behave in a comorbid setting can further exacerbate pulmonary complications associated with both disease states and impact susceptibility to respiratory infection. This review will provide a concise overview of how asthma distinctly affects individuals with sickle cell disease and how pulmonary physiology and inflammation are impacted during comorbidity.

## Introduction

Red blood cells (RBCs) constitute the largest number of mobile cells in the human body (about 3 × 10^12^) that perform the primary function of O_2_ (and CO_2_) transportation through hemoglobin (Hb). Alterations that occur in Hb through inherited genes can lead to a change in RBC morphology and function leading to sickle cell disease (SCD), a common inherited disorder leading to anemia, incidences of vaso-occlusive crises, acute chest syndrome (ACS), cumulative organ damage, and a number of additional chronic comorbidities (1). A large number of individuals carry the sickle cell trait, wherein a single sickle cell gene (“*S*”) is inherited, and are mostly asymptomatic (2). However, a patient with two sickle genes are named to have the Hb*SS* form of SCD, while a patient who inherits one *S* gene and another abnormal hemoglobin gene (C, beta thalassemia, D, E, or O) will have alternate types of SCD such as HbSC or HbS beta thalassemia. Patients with SCD represent a significant health care burden in terms of cost, and despite a number of therapeutic strategies, life expectancy in this population remains decades premature compared to that of the general population (3–5). As the most commonly inherited blood disease, SCD affects >100,000 in the United States and millions more worldwide (6). With 1:13 babies born with the sickle cell trait and 1:365 patients having SCD, African Americans have the highest incidence of SCD in the U.S. (7). The high occurrence of pulmonary complications in SCD patients has led to the consideration of possible complications from other respiratory conditions that have similar symptomatologies, like asthma.

Asthma is a syndrome of the respiratory system that affects 26 million Americans and 300 million globally. Like SCD, the incidence of asthma is predicted to continue to increase as indicated by the 3.6% increase in prevalence since 2006 (8). Of note is the observation that individuals with SCD have an increased incidence of asthma when compared to the general population. In children, the incidence of asthma diagnosis is as high as 27% in individuals with SCD (9). Approximately 30–70% of patients with SCD also suffer from asthma (10, 11) leading to a poorer quality of life. Like SCD, African Americans (especially women) are more likely to have asthma and African American children have a much higher likelihood of dying from asthma compared to other ethnicities (12). While it is unclear why asthma incidence is disproportionately elevated in African American children with SCD, socioeconomic factors and perhaps even overdiagnosis of asthma in SCD patients may contribute to this bias. ACS, one of the most frequent complications of SCD, is correlated with the incidence of asthma in the SCD population (13–15). As such, gaining an understanding of the clinical and immunological consequences of asthma in the context of SCD is of critical importance for improving patient outcomes in this patient group.

Asthma and SCD share a number of similarities in terms of the immunological factors associated with their respective disease states. Both conditions result in inflammation and airway hyperreactivity, both conditions impact susceptibility to respiratory infections, and both require specific interventions to mitigate the complications associated with them. Despite the recognition that asthma in the context of SCD likely results in a comorbid condition distinct from the general population, there is relatively little mechanistic insight into how these two disease pathologies co-function. In this review we highlight the potential immunological synergies between asthma and SCD garnered from both clinical data and murine modeling studies to showcase how these conditions may exacerbate each other, thereby representing a unique comorbid condition in these high-risk patient populations.

## Immunologic Consequences of Asthma in SCD

The immunologic sequelae associated with SCD and asthma are complex but have some overlap. Given that both asthma and SCD impact inflammation in distinct ways, the interplay into how these two conditions function when present in a comorbid state raises important questions. Elevated IgE levels in children with SCD is much more common than in the general population and is associated with both asthma and increased morbidity in children (9). Increased serum IgE is a well-accepted biomarker of allergic asthma, and SCD patients have elevated IgE in sera which may occur as a result of non-specific immune activation in these patients, leading to a T_H_2 bias and increased risk for asthma as a consequence. This enhanced serum IgE availability is also reflected in murine models, whereby the increase in total IgE in sensitized SCD mice is significantly greater than what is observed in sensitized wild type animals (16). Pulmonary function testing is often utilized to distinguish allergic asthma from other IgE mediated inflammatory conditions. Adult patients with SCD have a high incidence, up to 80%, of abnormal pulmonary function when tested (17). A similar, but less severe pattern is observed in children with SCD, with ~50% of patients having abnormal results (18). Abnormal results were more prevalant in the asthmatic pediatric SCD patients, underscoring applicability of pulmonary function analysis as part of making an appropriate diagnosis of asthma in SCD patients (18). The utility of screening for respiratory disorders such as asthma in children and adults using pulmonary function tests has not been fully established and current guidelines suggest routine collection of a thorough respiratory history to identify pulmonary disease in patients with SCD. This is of particular importance in young children because pulmonary function tests can be unreliable in this population. While asthma represents a major and frequent health concern for patients with SCD, the mechanistic factors driving the development and immunological features of asthma in the context of SCD remain poorly elucidated and create barriers to appropriate asthma management in SCD patients.

Endothelial activation is considered to be a major pathway by which sickled RBCs contribute to vaso-occlusion. Sickled RBC binding to integrins on endothelial cells lead to injury via reactive oxygen species that also function in a feed-forward loop to continue to activate endothelial cells (19). This activation leads to the infiltration of other cells such as monocytes and neutrophils which contribute to uncontrolled cell adhesion that occurs in blood vessels of SCD patients (20, 21). Increased levels of pro-inflammatory cytokines such as IL-3, GM-CSF, and PGE2 have also been noted to occur in SCD patients (22–24). Steady state levels of TNF-α, IL-α, IL-1β, and IL-6 are all elevated in SCD (23–25). Elevated neutrophil counts are characteristic of SCD and can form neutrophil extracellular traps in the pulmonary vasculature, contributing to acute lung injury resulting from inflammatory cytokine signaling (26). Both the pulmonary and systemic responses to inflammatory stimuli are greatly elevated in the context of SCD with enhanced levels of TNF-α, IL-1β, s-VCAM-1 being observed following endotoxin treatment (27). This heightened inflammatory landscape contributes to multiple complications of SCD, ranging from pulmonary disfunction and infection susceptibility.

**Figure 1:**
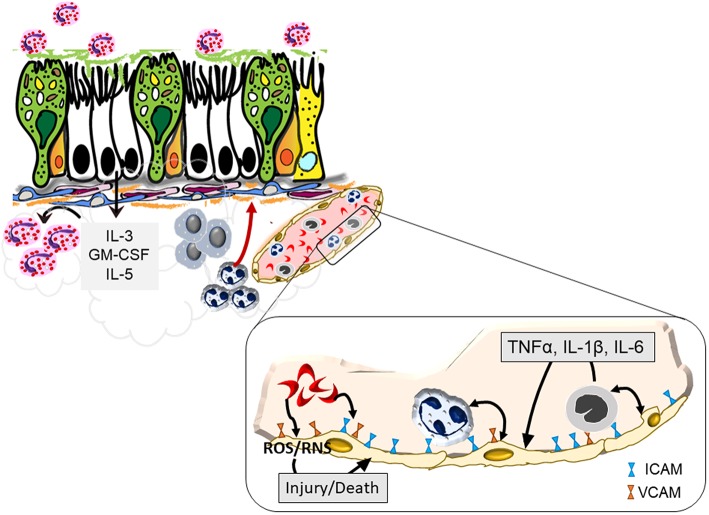
Endothelial activation by sickled red blood cells that may exacerbate asthma. Sickled red cells induce endothelial upregulation of integrins, ICAM, and VCAM, that enhance attachment and subsequent infiltration of neutrophils and monocytes into the pulmonary tissue. Increased margination of these leukocytes trigger further interaction with endothelial cells through the production of pro-inflammatory cytokines that together with sickled red blood cells cause endothelial cell production of reactive oxygen and nitrogen species that can trigger blood vessel injury. Recruited cells further activate the bronchial epithelium leading to a positive feedback loop to promote heightened inflammation and airway hyperreactivity.

Airway inflammation is a canonical hallmark of asthma and eosinophils may dominate as the infiltrating leukocyte in severe allergic asthma. Endothelial activation is fundamental to the initiation of inflammation in asthma (28) wherein endothelial cells upregulate integrins and selectins in response to allergenic stimuli (29) and cytokines produced *in situ* by resident leukocytes (30). Markers of endothelial activation including ICAM-1, VCAM-1, P-selection, and E-selectin are also elevated in the context of SCD (24, 31). Similarly, IL-3 and GM-CSF can promote the allergic milieu (32) and support activation and survival of eosinophils in the airways (33). While the exact role of PGE2 in the lungs of asthmatics is still unclear, its elevation correlates with eosinophilia (34). Common inflammatory pathways between SCD and asthma may therefore lead to an asthma-like phenotype in patients with SCD (Figure 1), or indeed, increase the likelihood of asthma pathogenesis in these patients.

## Modeling Asthma and SCD in Mice

While SCD is a hereditary condition, asthma development is dependent on genetic and environmental components. Although rodents do not naturally develop asthma, asthma-like disease can be triggered in them through continuous exposure to natural aeroallergens or ovalbumin (OVA) (35). The possible overlap between asthma and SCD based on shared symptoms such as airway inflammation, hyperresponsiveness, and architectural damage has created a demand for animal models of asthma and SCD comorbidity, although only a few have been created to date using OVA and house dust mite (HDM) as triggering allergens.

Existing models of asthma in SCD mice after OVA sensitization and challenge suggest that mice with SCD respond more severely to allergen exposures (16, 36, 37). OVA exposure leads to the development of peribronchovascular inflammation (with active eosinophils) and inflammatory foci, elevated serum IgE, and bronchial epithelial hyperplasia in BERK SCD mice to equivalent levels as wild-type controls (16). However, more severe pathologic changes occur in SCD mice when OVA-challenge duration is prolonged causing death in about 30% of the animals (16), suggesting that the BERK SCD mice may have a lower threshold for asthma exacerbation. Using a bone marrow chimeric mouse model of SCD and shortened exposures to aerosolized OVA, Pritchard et al. demonstrated that OVA-induced allergic inflammation in these SCD mice correlates with a heightened T_H_2 cytokine milieu and pulmonary tissue resistance marked by a decrease in lung tissue elasticity suggestive of greater alveolar occlusion (36). This trend in airway inflammation and general T_H_2 skewing was recapitulated by Andemariam et al. using the BERK mouse model of SCD and a more standard model of OVA exposure (37). Of note, naïve BERK SCD have increased levels of T_H_2-type cytokines in the bronchoalveolar lavage fluid and a higher number of T-lymphocytes in the lungs (37). Airway hyperresponsiveness is a shared hallmark of SCD and asthma (38, 39), that generally correlates with pulmonary inflammation. Despite heightened airway inflammation in response to OVA, BERK SCD mice had lower airway reactivity compared to wild-type mice even at very high doses of methacholine (37), suggesting that inflammation and airway hyperresponsiveness may be disjointed in SCD.

Although OVA is a commonly used trigger to induce allergic asthma-like disease in mice, due to its limitations as a clinically relevant aeroallergen, utilization of more relevant allergens such as HDM, cockroach, fungal, and viral antigens have gained popularity among investigators that model asthma in mice (40). Most recently, Jiang et al. found no differences between BERK SCD and wild-type mice in the inflammatory index, airway cytokines, or HDM-specific IgE levels after HDM exposure (41). These findings are exciting as they confirm that variation in outcome occurs based on the antigen, route of exposure, and adjuvants in SCD mice. Mouse models of SCD with asthma that can be used to decipher mechanisms that underlie asthma pathogenesis in patients with SCD is an important gap in technology to address.

## Treatment of Asthma in Individuals with Sickle Cell Disease

Information amassed on the immunologic basis of asthma has resulted in the development of biologics targeted for patients with specific endotypes. However, since the efficacy of these therapeutics are quite low, corticosteroids are used to alleviate the symptoms during asthma attacks despite our knowledge of the long term negative impact of steroids on human health (42). Although the SCD-asthma comorbid condition is prevalent, relatively little evidence based models exist for its management. Current models for asthma management in SCD are based on NIH guidelines for the general population and include liberal use of inhaled steroids, despite the extensive literature recognizing SCD patients as a uniquely susceptible and vulnerable patient population (10, 43, 44). Use of inhaled steroids further increases the risk of colonization of *S. pneumoniae* which may increase the likelihood for the development of invasive disease to which the SCD population is particularly susceptible (45, 46). Inhaled corticosteroids have been proposed to be used to prevent additional episodes of vaso-occlusive crisis in pediatric patients, and recent studies have underscored the feasibility of this approach in young children (47). Inhaled steroids given to non-asthmatic patients with SCD have demonstrated considerable promise, with significant reductions in pain and sVCAM levels as well as inflammatory macrophage markers, underscoring the potential for targeting inflammation to improve health outcomes in these patients (48–50). Whether treatments for specific asthma endotypes can be extended to patients with SCD remains unclear, though given the underlying differences in inflammation tailored therapeutic strategies may be required.

## SCD and Asthma: Independent Pathways to Infection Susceptibility

Asthma and SCD both fundamentally alter susceptibility and immune responses to respiratory infection. Patients with SCD are overwhelmingly susceptible to multiple respiratory pathogens, most importantly the pneumococcus (51, 52), and infectious diseases increase the development of ACS in these patients (53–55). The heightened sensitivity to infection is recapitulated in the murine model of SCD, whereby the SCD mice demonstrate dramatically enhanced susceptibility to both bacterial and viral respiratory infections (56–58). This issue can be further confounded by strains outside of vaccine coverage causing invasive disease in these patients, as is the case with *S. pneumoniae* (59). Immunogenic responses to vaccines in SCD patients may also be suboptimal to confer effective protection as the responses have been reported to wane more rapidly to a number of serotypes included in the current pneumococcal vaccines, an observation that has been recapitulated in murine models of SCD (60, 61). These underscore the importance of appropriate prophylactic strategies to mitigate infection risk in individuals with SCD.

Similar but distinct to what is observed in the context of SCD, allergic asthma dramatically also alters susceptibility to multiple respiratory pathogens including both viral and bacterial pathogens (62). However, unlike in the case of SCD, allergic asthma has been found to confer both sensitivity and resistance to subsequent respiratory infection, wherein outcomes are more likely to be dependent on the type of pathogen. Asthma exacerbations triggered by rhinoviruses and respiratory syncytial virus, for example, can be detrimental to the host (63), while asthma exacerbations triggered by influenza virus infection is tolerated by the host which also exhibit reduced signs of influenza morbidity and enhanced viral clearance (64, 65). Immune responses to viruses in hosts with asthma may be dependent on a multitude of factors including gender, age, virus strain and prior exposures, endotype of asthma, and environmental factors including pollution and nutrition. How these alterations in pulmonary inflammation during the asthma-SCD comorbid state differentially impact the risk of infection remains poorly understood, though given the distinct nature of these two disease settings, it may be anticipated that together they impact infection susceptibility in a manner distinct from the general population.

## Antibiotic Exposure and Asthma

Due to the propensity of patients with SCD to develop fulminant lethal sepsis caused by *S. pneumoniae*, during childhood, penicillin prophylaxis is prescribed for all children with SCD until the age of 5 years, which has dramatically improved mortality in this patient population prior to the advent of the pneumococcal conjugate vaccine (66–70). There is considerable evidence linking antibiotic exposure to the development of childhood asthma and other allergic disease in the general population, though there are challenges in terms of confounding respiratory infections (71–79). Early antibiotic use is associated with allergic asthma in young children even when accounting for bias inherent from antibiotic prescriptions to treat early symptoms of asthma; this is predictable as bacterial colonization of the respiratory and gastrointestinal tract are critical mediators that shape susceptibility to allergic airway inflammation (80–82). These effects may be amplified in SCD patients due to the prolonged exposure to antibiotics.

Administering penicillin to patients with SCD eliminates several bacterial species from the nasal-oral microbiota (83). Exposure to antibiotics early in life can have long lasting consequences on the developing bacterial microbiome (84, 85). Bacterial colonization of the respiratory and gastrointestinal tract are critical mediators that can shape susceptibility to allergic airway inflammation (82). Numerous gaps in knowledge including alterations that may naturally occur in the microbiome of the SCD host (86), the relative impact of long-term penicillin prophylaxis on the intestinal and respiratory flora of these patients, and whether this long-term prophylaxis also impacts the likelihood of subsequent asthma development preclude our understanding of disease pathogenesis in SCD patients and those that may develop asthma.

## Arginine Deficiency: A Common Crossroad in SCD and Asthma Pathophysiology

Arginine deficiency has long been recognized as an important aspect of SCD pathophysiology (87, 88). Low arginine bioavailability is associated with a multitude of complications in SCD including pulmonary hypertension and vaso-occlusive pain episodes (89–91). The decreased arginine availability in SCD is severe enough to impact the contribution of bacterial arginine biosynthesis and uptake pathways to virulence (57). Due to the multiple facets of host pathophysiology, arginine supplementation has been proposed as a therapeutic intervention for SCD (92, 93). Arginine supplementation has been suggested as a means by which to alleviate complications in patients with SCD and improve overall health (94, 95). Clinical trials further support the potential for arginine supplementation to confer benefit in SCD individuals in terms of endothelial function and to induce nitric oxide production during vaso-occlusive crisis (93, 96).

**Figure 2:**
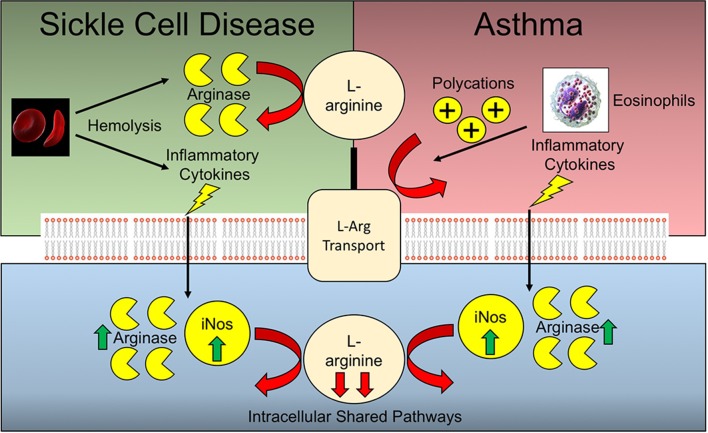
Arginine deficiencies in Sickle cell disease and asthma. Sickle cell disease and asthma share complementary and potentially synergistic mechanisms of arginine deficiency. Many of the pathways operative extracellularly are different, while the intracellular pathways are shared.

Arginine deficiency is a common feature underlying the pathophysiology of both allergic asthma and SCD (Figure 2). Murine models of allergic asthma have demonstrated that arginine deficiency to nitric oxide synthase (NOS) results in deficiencies in nitric oxide, a bronchodilator, in tandem with increased peroxynitrite, a pro-contractile molecule, both of which contribute to airway hyperresponsiveness in the context of asthma. Polycation secretion by eosinophils, which are dramatically elevated in allergic asthma, can inhibit arginine uptake via the y+ system (97, 98). In the context of SCD, there are additional mechanisms underlying arginine deficiency that are independent of pathways operative during asthma. The increased hemolysis of RBCs leads to the release of cellular arginase which can scavenge arginine prior to cellular uptake. In the context of asthma and SCD comorbidity, heightened baseline inflammation may lead to increased expression of both iNOS and arginase, thereby further depleting cellular arginine pools (99). As such, the extracellular arginase released by hemolysis coupled with the increased pulmonary eosinophil infiltrate inhibiting arginine uptake are likely to have an additive effect. Likewise, the increased arginase and iNOS activity resulting from increased levels of inflammatory cytokines inherent in both SCD and allergic asthma, are also potentially synergistic in terms of arginine depletion. Due to the divergence of many of these arginine depleting pathways, it would be expected that such deficiency may be synergistic in comorbid patients with SCD and asthma.

The benefits conferred by arginine supplementation may be most evident in comorbid patients with both SCD and asthma due to the non-overlapping mechanisms of arginine deficiency.

## Discussion

SCD and asthma share similar manifestations in terms of airway hyperreactivity despite being immunologically distinct diseases. Experimental modeling and clinical data suggest that asthma impacts individuals with SCD in a specific manner distinct from the general population. Laying a mechanistic foundation for understanding pulmonary complications of sickle cell disease and how these complications can be rationally targeted in a SCD-specific manner may provide novel opportunities for treatment. Given the unique host pathophysiology that underlies SCD, these individuals may benefit from tailored interventions for the treatment of asthma.

## Author Contributions

JR and AS wrote the manuscript jointly.

### Conflict of Interest

The authors declare that the research was conducted in the absence of any commercial or financial relationships that could be construed as a potential conflict of interest.
